# Evidence‐based Protocols for reducing the probability of Alzheimer's Desease in older adults: Correlational insights in Chilean Study

**DOI:** 10.1002/alz70858_099028

**Published:** 2025-12-24

**Authors:** Christine Gierke, Carlos F. Navarro, Melissa Martinez, Carolina Delgado, Mauricio Cerda, Walthers Diaz‐Gierke, Gonzalo Farías

**Affiliations:** ^1^ Los Andes University, Santiago, Chile; ^2^ University of Chile, Santiago, Chile; ^3^ J.J. Aguirre's Clinical Hospital, University of Chile, Santiago, Chile; ^4^ Silva Henriquez Catholic University, Santiago, Chile; ^5^ San Sebastián University, Santiago, Chile; ^6^ University of Chile, Biomedical Neuroscience Institute (BNI), ICBM, Santiago, Chile; ^7^ J.J. Aguirre's Clinical Hospital, University of Chile, Santiago, Chile; ^8^ Universidad de Chile, Santiago, Santiago, Chile; ^9^ Hospital Clínico Universidad de Chile, Departamento de Neurología y Neurocirugía, Santiago, Santiago, Chile; ^10^ University of Chile, Biomedical Neuroscience Institute (BNI), ICBM, Santiago, Metropolitana, Chile; ^11^ CIMT Center for Medical Informatics and Telemedicine, School of Medicine, Universidad de Chile, santiago, Chile; ^12^ Geroscience Center for Brain Health and Metabolism (GERO), Santiago, Chile; ^13^ Adolfo Ibañez University, Santiago, Chile; ^14^ J.J. Aguirre's Clinical Hospital, University of Chile, Santiago, Chile

## Abstract

**Background:**

Depression is a significant public health among older adults, with distinct manifestations and risk factors influenced by sex. Our study on post‐pandemic social isolation and loneliness in a cohort of chilean older adults revealed critical sex‐specific correlations. Women showed stronger associations between loneliness, social isolation (*p* = <0.001), depression (*p* = 0.005) and pandemic‐related concern (*p* = 0.481), while men exhibited notable links between Age and social isolation (*p* = <0.001) and negative associations with Loneliness and pandemic related concerns (*p* = 0.127) (Gierke, C., et al., 2025). These findings highlight the need for tailored interventions addressing the unique psychosocial dynamics between older adults.

**Methods:**

Based on our findings, we analyzed psychosocial and demographic variables, including social isolation, loneliness, level of concern and depressive symptoms. A ***sex‐sensitive therapeutic protocol*** was developed to guide therapeutic strategies, emphasizing emotional, cognitive, and social engagement tailored to the unique needs of each group.

**Results:**

This proposal suggests that for women's depression was closely linked to heightened emotional reactivity to loneliness and concern. Interventions prioritizing emotional support, peer networks, and resilience training were identified as critical. Group therapy, mindfulness‐based interventions, and intergenerational programs showed promise for mitigating depressive symptoms. For Men, depression was associated with reduced social networks post‐retirement and age‐related changes. Structured, goal‐oriented interventions, such as volunteer programs, mentorship opportunities, and skill‐based group activities, were found to be effective. These strategies address men's instrumental coping styles and encourage rebuilding of social networks.

**Conclusion:**

Sex‐specific therapeutic strategies are essential for effectively managing risk factors for Alzheimer's Desease in older adulta. For women addressing emotional needs through connection and support is key, while for men, interventions should focus on fostering purpose and rebuilding social roles. These tailored approaches not only reduce depressive symptoms but also enhance overall well‐being contributing to the prevention of long‐term health risks such as cognitive decline and dementia. Integrating these findings into public health initiatives can pave the way for more impactful mental health interventions.

